# Improving management of glycaemic control in people with T2DM in primary care: estimation of the impact on the clinical complications and associated costs

**DOI:** 10.1186/s12913-020-05360-w

**Published:** 2020-08-26

**Authors:** M. Mata-Cases, J. Mahon, D. Mauricio, J. Franch-Nadal, J. Real, N. Hex

**Affiliations:** 1 DAP-Cat group, Unitat de Suport a la Recerca Barcelona Ciutat, Institut Universitari d’Investigació en Atenció Primària Jordi Gol (IDIAP Jordi Gol), CIBER of Diabetes and Associated Metabolic Diseases (CIBERDEM), Instituto de Salud Carlos III (ISCIII), Carrer Sardenya 375, entlo 1ª, 08025 Barcelona, Spain; 2 York Health Economics Consortium Ltd, University of York, York, UK

**Keywords:** Type 2 diabetes, Health economics, Diabetes complications, Glycaemic control

## Abstract

**Background:**

To estimate the potential benefits in terms of avoided complications and cost reduction if the Spanish health system would encourage the intensification of treatment for better glycaemic control in adults with Type 2 diabetes from the current HbA1c target used in clinical practice of 68 mmol/mol to a target of 53 mmol/mol.

**Methods:**

The IQVIA Core Diabetes Model (version 9.0) was used to model the impact of these changes in respect of micro- and macrovascular complications and the associated costs. The modelling was based on data derived from the SIDIAP-Q population database from Catalonia, taking a random cohort of 10,000 people with type 2 diabetes and dividing it into sub-groups based on their baseline HbA1c.

**Results:**

The CDM modelling showed that the average cost reduction per person varies depending on baseline HbA1c. The model estimates that after 25 years, people with a baseline HbA1c between 48 and 58 mmol/mol and > 75 mmol/mol show an average cost reduction of €6027 and €11,966, respectively. Applying the per-person cost reduction to the cohorts of the prevalent population in Spain (1,910,374) the overall estimated cost reduction was €14.7 billion over 25 years. The improvements in outcomes resulted in an estimated reduction of more than 1.2 million complications cumulatively over 25 years, of which more than 550,000 relate to diabetic foot and more than 170,000 related to renal disease.

**Conclusion:**

Over a 25 year period, Spain could considerably reduce costs and avoid major complications if, on a population level, more ambitious glycaemic control, according to Spanish or EU guidelines, could be achieved among people with type 2 diabetes by reducing the HbA1c threshold for treatment intensification. Although there is a slower trajectory for benefits in earlier years, there is a much more rapid benefit gain between years 5 and 15.

## Background

Type 2 diabetes incurs a very high cost burden for health care systems and individual organizations, both in relation management of the disease and its associated complications [1–4]. This is the case for both primary and specialist care organizations and is especially pertinent in countries with a National Health System, which are generally encountering significant financial pressures. Some studies have shown that the healthcare costs of people with diabetes can be 60 to 80% higher than for people without diabetes [2–4]. Much of this additional cost burden can be accounted for by late complications such as end stage renal disease (ESRD), which because it requires dialysis and transplantation, can result in more significant cost increases than those relating to, for example, cardiovascular disease [1, 5]. Complications such as ESRD also have a more significant impact on morbidity and mortality [1, 5].

Glycaemic control statistics show that in Spain, although population-wide diabetes management is acceptable or similar to other countries, it could be improved [6–8]. In general, intensification of Type 2 diabetes treatment is often delayed resulting in poor control and increased risk of costly and preventable complications. As in other countries, current evidence is that the mean HbA1c level of treatment intensification is higher than clinically recommended. According to four different studies, treatment intensification in Spain, was made around 68 mmol/mol [7–10]. In 2011, the way HbA1c values were reported switched from a percentage to a measurement in millimoles per mole (mmols/mol). 68 mmol/mol equates to 8.4% and 53 mmol/mol equates to 7.0%.

The clinical practice guideline for the Spanish National Health System (NHS) on type 2 diabetes states that whilst individual patient clinical needs and preferences should be taken into account, a target of 53 mmol/mol for HbA1c should be considered desirable with treatment adjusted accordingly [11]. This is in accordance with the European Society of Cardiology Guidelines on HbA1c targets [12]. If diet and exercise should fail to keep HbA1c to the target then the treatment algorithm in the guideline recommends to start a patient on monotherapy, then to progress to a combination therapy (dual or triple) and finally to insulin. Treatment intensification should be considered when the HbA1c target of 53 mmol/mol is not being achieved. Nevertheless, in Spain, studies conducted in primary care reported that the lack of intensification in people with diabetes with poor glycemic control (HbA1c ≥53 mmol/mol) varies between 32.2 and 52.5% [7, 13, 14]. The longest delays in treatment intensification are known to occur among people with diabetes on two or more non-insulin anti-diabetic drugs [10, 14, 15].

The glycated hemoglobin (HbA1c) value at which treatment intensification is recommended varies between 48 mmol/mol and 64 mmol/mol in international and national guidelines, and it is 64 mmol/mol for incentivization purposes in our institution (Catalan Institute of Health; ICS) [16]. This national threshold is based on the idea that people with HbA1c levels above 64 mmol/mol are the most likely to benefit from a timely intensification and to avoid overtreatment in the elderly, but the evidence on the cost-effectiveness of this approach is scarce.

Baxter et al. (2016) reported that in the UK, improvements in glycaemic control led to an average reduction and delay in the onset of all diabetes-related complications and mortality rates [17]. The cumulative cost reduction for the UK health system over 25 years was estimated at almost £5 billion for Type 2 diabetes if people with diabetes receive treatment escalations according to the National Institute of Health and Care Excellence (NICE) Type 2 diabetes treatment algorithm [18].

The aim of this study was to use a similar approach to that used by Baxter et al. to estimate the effect of improvements in glycaemic control on the cumulative rate of type 2 diabetes complications in Spain and the associated costs. Specifically, the main objective was to examine the impact of intensifying treatment to better control HbA1c at the target of 53 mmol/mol, instead of the current mean HbA1c level of treatment intensification (68 mmol/mol). The study does not incorporate any costs related to interventions that could be used to intensify treatment because it is for individual health commissioners and providers to determine the interventions they wish to invest in to improve glycaemic control in people with Type 2 diabetes and care should be individualised for each person. The purpose of the study is to highlight the potential that might be available for investment in diabetes care models and programmes rather than to demonstrate the outcome of a specific intervention or set of interventions.

## Methods

Modelling simulations were carried out to examine the impact of intensifying treatment to better control HbA1c at the target of 53 mmol/mol, instead of the current mean HbA1c level of treatment intensification (68 mmol/mol). Clinical guidelines, such as the Spanish clinical practice guideline for diabetes and the ESC guideline [11, 12], suggest treatment algorithms that aim to maintain HbA1c at or below target levels in order to achieve optimal outcomes for people with diabetes.

The IQVIA Core Diabetes Model (CDM) is able to run patient-level modelling simulations in this way. The CDM is a widely published and validated [19, 20] model for Type 1 and Type 2 diabetes. It is a non-product specific computer simulation model designed to translate surrogate endpoints into long-term health and economic outcomes http://www.core-diabetes.com/.

The CDM includes interdependent sub-models that simulate changes in the rates of microvascular and macrovascular complications and mortality associated with diabetes, for different management strategies. The CDM is populated with demographic data on a cohort of people with diabetes, costs (the ‘economic setting’), treatment values for risk factors and adverse events, a treatment algorithm, disease management characteristics and clinical settings. The model structure comprises 17 interdependent sub-models that simulate the complications of diabetes over a time period, in this case, of 25 years. The model is a fixed-time increment (annual) stochastic simulation with each sub-model using time, state, and diabetes-type dependent probabilities. The CDM uses transition probabilities and management strategies Type 2 diabetes, with the predominant sources of data being the UKPDS studies [19]. Table 1 shows the management and complication costs used.

**Table 1:** Management and complication costs used in the CDM

**Management costs**	**Cost (€)**	**Source**
Statins (Atorvastina 20 MG 28 comprimidos)	120.14	https://botplusweb.portalfarma.com/botplus.aspx Botplus 2015
Aspirin (Adiro 100 mg 30 comprimidos)	88.27	https://botplusweb.portalfarma.com/botplus.aspx Botplus 2015
ACE inhibitor (Enalapril 20 MG 28 comprimidos)	21.00	https://botplusweb.portalfarma.com/botplus.aspx Botplus 2015
Annual Screening for microalbuminurea	50.83	eSalud 2015 (official tariff)
Annual screening gross renal protenuira	203.44	eSalud 2015 (official tariff)
Stopping ACEs due to side effects	54.08	[21] Fonseca 2013
Annual eye screening	55.27	eSalud 2015 (official tariff)
Foot screening program	23.69	eSalud 2015 (official tariff)
Non-standard ulcer treat (eg. Regranex)	–	
Anti-depression treatment	288.05	[22] Salvador-Carulla 2009
Screening for depression	–	eSalud 2015 (official tariff)
**Direct costs CVD complications**	**Cost (€)**	**Source**
Myocardial infarction 1st year average	5282.63	[23] Abad 2015
Myocardial infarction 1st year fatal	4566.21	[23] Abad 2015
Myocardial infarction 1st year non-fatal	5383.13	[23] Abad 2015
Myocardial infarction 2nd + years	860.43	[23] Abad 2015
Angina 1st year	2373.60	[23] Abad 2015
Angina 2nd + years	860.43	[23] Abad 2015
Congestive heart failure 1st year average	3564.34	[23] Abad 2015
Congestive heart failure 1st year fatal	4566.21	[23] Abad 2015
Congestive heart failure 1st year non-fatal	3505.02	[23] Abad 2015
Congestive heart failure 2nd plus years	3554.47	[23] Abad 2015
Stroke 1st year non-fatal	6658.42	[23] Abad 2015
Stroke 2nd + years	3595.02	[23] Abad 2015
Stroke death within 30 days	4566.21	[23] Abad 2015
Peripheral vascular disease 1st year	2373.60	[23] Abad 2015
Peripheral vascular disease 2nd + years	860.43	[23] Abad 2015
**Direct costs renal complications**	**Cost (€)**	**Source**
Haemodialysis 1st year	47,069.14	[24] Villa 2011
Haemodialysis 2+ years	43,997.16	[24] Villa 2011
Peritoneal dialysis 1st year	32,022.17	[24] Villa 2011
Peritoneal dialysis 2+ years	29,927.06	[24] Villa 2011
Renal transplant costs 1st year	51,677.66	[24] Villa 2011
Renal transplant 2+ years	7280.71	[24] Villa 2011
**Direct costs acute events**	**Cost (€)**	**Source**
Major hypoglycaemic event	1038.23	[25] Hammer 2009
Minor hypoglycaemic event	59.07	[25] Hammer 2009
Lactic acid event	3446.53	Ministry of Health 2012
Edema onset (adverse event)	5225.52	Ministry of Health 2012
Edema follow-up (adverse event)	–	
**Direct costs acute events**	**Cost (€)**	**Source**
Laser treatment	126.84	eSalud 2015 (official tariff)
Cataract operation	1006.63	eSalud 2015 (official tariff)
Following cataract operation	52.32	Tarifas CCAA 2008.
Blindness - year of onset	1875.14	[23] Abad 2015
Blindness - following years	805.05	[23] Abad 2015
**Direct costs acute events**	**Cost (€)**	**Source**
Neuropathy, 1st year	4653.06	[26] Rodríguez 2011
Neuropathy, 2nd + years	4653.06	[26] Rodríguez 2011
Amputation (event based)	3644.47	[23] Abad 2015
Amputation Prosthesis (event based)	3712.05	Estimation
Gangrene treatment	10,131.19	eSalud 2015 (official tariff)
After healed ulcer	–	
Infected ulcer	5042.78	eSalud 2015 (official tariff)
Standard uninfected ulcer	1120.96	eSalud 2015 (official tariff)
Healed ulcer history of amputation	–	

To populate the CDM a representative cohort of patient-level data were drawn from the SIDIAP-Q database of 126,811 people with type 2 diabetes, cared for by the Catalonian Health Institut in 2011 in Catalonia. Although Catalonia may have different demographic characteristics to the rest of Spain, the patients in the SIDIAP-Q database can be considered a typical cohort of people with diabetes cared for in primary care.

SIDIAP (Information System for the Development of Research in Primary Care) is a database of electronic medical records started in 2006 http://www.sidiap.org/index.php/en. The SIDIAP-Q subpopulation is composed of those people with the most complete medical histories and includes data from 1,878,816 of the 5.8 million patients registered in the parent SIDIAP database.

Methodological details of the study of the cost of type 2 diabetes mellitus using this database have been described in a previous publication [4]. SIDIAP holds longitudinal patient information which is anonymized and drawn from primary care centres in Catalonia using the electronic clinical station for primary care (eCAP). The database includes the medical records of all patients cared for by the Catalan Health Institute and for investigation purposes provides anonymized information on socio-demographic characteristics, health problems using International Classification of Diseases codes (ICD-10), detailed clinical markers, lifestyle measures, diagnostic and clinical procedures, specialist referrals, the results of laboratory and all electronically prescribed treatments. The CatSalut (Catalan National Health Service) general database provides additional data on hospitalization (diagnosis, procedures and length of stay) and the pharmacological treatments actually billed to the CatSalut.

The cohort of people modelled in the CDM was made up of a sample of 10,000 people in the SIDIAP-Q database, selected randomly into four sub-groups of 2500 based on their baseline level of HbA1c: (48 to ≤58 mmol/mol, 58 to ≤64 mmol/mol; 64 to ≤75 mmol/mol; and > 75 mmol/mol). Table 2 shows the patient cohort characteristics. For each of the HbA1c groups, estimates of future outcomes were produced using CDM (v9.0) to predict the cumulative incidence of complications for the base case (HbA1c treatment escalations at the current 68 mmol/mol) and for the comparator case (HbA1c escalations at 53 mmol/mol).

**Table 2:** Patient cohorts characteristics segmented by HbA1c range

	All cohorts (*N* = 10,000)	Cohort 1 48 to ≤58 mmol/mol (*N* = 2500)	Cohort 2 58 to ≤64 mmol/mol (*N* = 2500)	Cohort 3 64 to ≤75 mmol/mol (*N* = 2500)	Cohort 4 > 75 mmol/mol (*N* = 2500)
Age [years (SD)]	68.1 (11.5)	68.4 (11.6)	69.1 (10.8)	68.9 (10.9)	65.8 (12.2)
Sex (women)	4811 (48.1%)	1158 (46.3%)	1250 (50.0%)	1260 (50.4%)	1143 (45.7%)
Diabetes duration [years (SD)]	7.33 (5.89)	5.97 (5.26)	6.56 (5.21)	8.07 (6.20)	8.73 (6.38)
BMI [kg/m^2^ (SD)]	30.5 (5.18)	30.4 (5.12)	30.4 (4.92)	30.3 (5.35)	30.8 (5.30)
Obesity	4510 (48.2%)	1082 (46.9%)	1141 (48.5%)	1075 (45.4%)	1212 (52.0%)
Hypertension	7102 (71.0%)	1794 (71.8%)	1794 (71.8%)	1842 (73.7%)	1672 (66.9%)
Smoking habit					
Never smoker	6177 (63.4%)	1535 (63.7%)	1581 (64.8%)	1594 (64.9%)	1467 (60.1%)
Current smoker	1358 (13.9%)	323 (13.4%)	294 (12.1%)	322 (13.1%)	419 (17.2%)
Ex-smoker	2207 (22.7%)	551 (22.9%)	563 (23.1%)	540 (22.0%)	553 (22.7%)
SBP [mmHg (SD)]	137 (13.4)	136 (13.3)	136 (13.1)	137 (12.8)	139 (14.0)
DBP [mmHg (SD)]	76.3 (8.42)	75.7 (8.43)	76.0 (8.11)	76.1 (8.29)	77.4 (8.76)
LDL Cholesterol [mg/ml (SD)]	111 (32.8)	112 (32.2)	112 (32.4)	110 (31.0)	113 (35.5)
Chronic complications					
Microalbuminuria	793 (14.1%)	144 (11.1%)	164 (11.7%)	195 (13.3%)	290 (20.0%)
Macroalbuminuria	131 (2.33%)	24 (1.86%)	25 (1.78%)	24 (1.64%)	58 (3.99%)
Chronic renal failure [(eGFR< 60 ml/min (SD)]	1593 (18.5%)	414 (20.2%)	401 (18.2%)	424 (19.4%)	354 (16.3%)
Retinopathy	751 (7.51%)	119 (4.76%)	129 (5.16%)	203 (8.12%)	300 (12.0%)
Neuropathy	924 (16.5%)	168 (13.0%)	189 (13.4%)	219 (15.0%)	348 (24.0%)
Stroke	764 (7.64%)	196 (7.84%)	179 (7.16%)	199 (7.96%)	190 (7.60%)
Coronary Heart Disease	1280 (12.8%)	301 (12.0%)	300 (12.0%)	314 (12.6%)	365 (14.6%)
Heart Failure	555 (5.55%)	159 (6.36%)	110 (4.40%)	134 (5.36%)	152 (6.08%)
Peripheral arteriopathy	477 (4.77%)	109 (4.36%)	95 (3.80%)	125 (5.00%)	148 (5.92%)
Any macrovascular	2497 (25.0%)	619 (24.8%)	565 (22.6%)	630 (25.2%)	683 (27.3%)
Coexistence of complications:					
No complications	6408 (64.1%)	1698 (67.9%)	1723 (68.9%)	1572 (62.9%)	1415 (56.6%)
only macrovascular	867 (8.67%)	250 (10.0%)	196 (7.84%)	220 (8.80%)	201 (8.04%)
only microvascular	1095 (10.9%)	183 (7.32%)	212 (8.48%)	298 (11.9%)	402 (16.1%)
both	1630 (16.3%)	369 (14.8%)	369 (14.8%)	410 (16.4%)	482 (19.3%)
Steps of antidiabetic treatment					
Life-style only	2286 (22.9%)	1042 (41.7%)	663 (26.5%)	358 (14.3%)	223 (8.92%)
Non insulin Antidiabetics	6015 (60.2%)	1333 (53.3%)	1630 (65.2%)	1693 (67.7%)	1359 (54.4%)
Insulin	1699 (17.0%)	125 (5.00%)	207 (8.28%)	449 (18.0%)	918 (36.7%)

This simulated the effect of a management strategy for type 2 diabetes that intensified or escalated treatment when people reached a threshold of 53 mmol/mol compared to current practice whereby treatment is intensified at 68 mmol/mol on average. The effect of each escalation of treatment on the development of complications was modified by the duration of disease and a maximum of five treatment escalations were considered within a 25 year period. It was assumed that each escalation of treatment would be associated with a reduction in HbA1c level by 11 mmol/mol [27]. The UK Prospective Diabetes Study (UKPDS) recorded a 37% reduction in microvascular complications for an 11 mmol/mol reduction in HbA1c [28]. No discounting was applied to the costs estimated as this was a budget impact analysis rather than a cost-effectiveness analysis.

Specific forms of treatment that would lead to an 11 mmol/mol reduction in HbA1c were not detailed in the analysis because different patients would require different forms of escalation depending on the progression of their disease. The better management strategy should be viewed not as a treatment itself, but rather a collection of activities to encourage people with diabetes and clinicians to maintain or achieve lower HbA1c levels. For those with a higher baseline level of HbA1c, the modelling allowed several successive treatment escalations (up to five times) in order to bring HbA1c levels to target levels.

## Results

The SIDIAP-Q database includes 126,811 people in the Catalunya region with type 2 diabetes over the age of 30 in 2011. Of the people with a recorded HbA1c level (93,351), 34.23% have an HbA1c starting level below 48 mmol/mol; 34.8% are between 48 mmol/mol and 57 mmol/mol; 10.07% are between 58 mmol/mol and 63 mmol/mol; 10.96% are between 64 mmol/mol and 74 mmol/mol; and 9.94% are greater than 75 mmol/mol.

The prevalence of known diabetes in Spain is estimated at 7.8% [29] and the population older than 18 in Spain is 37.2 million [30]. Within the SIDIAP-Q database, 65.77% of people with a recorded level had HbA1c > 48 mmol/mol. On that basis, we have estimated the adult population of people identified with diabetes and with HbA1c > 48 mmol/mol is 1,910,374. The proportions of people in each of the cohorts in the SIDIAP database were used to estimate the total numbers of people with starting HbA1c levels in each cohort for the whole of Spain.

The CDM modelling showed that for these cohorts of people with diabetes, if treatment is escalated at HbA1c of 53 mmol/mol instead of current practice (treatment escalation at 68 mmol/mol), the average cost reduction per person varies depending on their baseline HbA1c. After five years, people with starting HbA1c of > 48 mmol/mol to ≤58 mmol/mol show an average cost reduction of €198, while those with starting HbA1c of > 75 mmol/mol showed an average cost reduction of €1588 per person. These increased to €6027 and €11,966 per person respectively after 25 years. As mentioned previously, the cost reduction estimates do not include any estimates of the costs of interventions to improve glycaemic control. Table 3 shows the cost reductions per person and for the population.

**Table 3:** Cost reductions for avoided complications for the Spanish type 2 diabetes population with starting HbA1c > 48 mmol/mol

**Adult type 2 diabetes per-person cost reductions**					
	**5 years**	**10 years**	**15 years**	**20 years**	**25 years**
> 48 to ≤58 mmol/mol	€198	€1754	€3653	€5221	€6027
58 to ≤64 mmol/mol	€548	€2492	€4563	€6108	€6633
64 to ≤75 mmol/mol	€1569	€4249	€6892	€9039	€10,233
> 75 mmol/mol	€1588	€4529	€7647	€10,369	€11,966
**Adult type 2 diabetes total population cost reductions**					
	**5 years**	**10 years**	**15 years**	**20 years**	**25 years**
> 48 to ≤58 mmol/mol	€200,133,593	€1,772,900,617	€3,692,363,715	€5,277,260,048	€6,091,945,280
58 to ≤64 mmol/mol	€160,234,152	€728,656,033	€1,334,212,472	€1,785,967,517	€1,939,476,513
64 to ≤75 mmol/mol	€499,489,460	€1,352,664,574	€2,194,060,778	€2,877,555,916	€3,257,664,530
> 75 mmol/mol	€458,695,151	€1,308,205,503	€2,208,842,455	€2,995,094,470	€3,456,389,279
**TOTAL**	**€1.319 bn**	**€5.162 bn**	**€9.429 bn**	**€12.936 bn**	**€14.745 bn**

Applying the per-person cost reduction to the cohorts of the prevalent population gives an overall estimated cost reduction of €14.7 billion over 25 years. The estimated overall cost reduction rises from €1.3 billion in year 5 to €5.2 billion in year 10, to €9.4 billion in year 15 and to €12.9 billion in year 20.

**Fig. 1:**
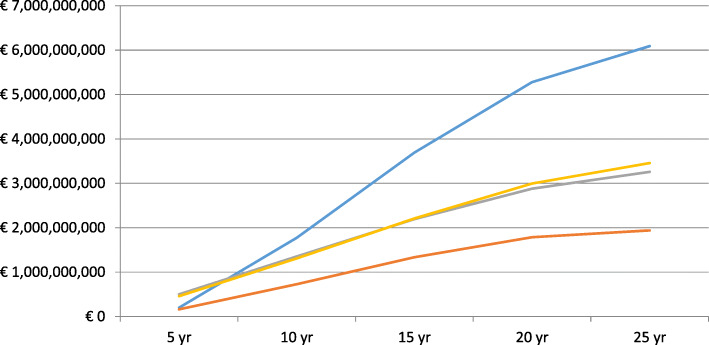
Cumulative cost reduction for the Spanish type 2 diabetes population with starting HbA1c > 48 mmol/mol. Blue = > 48 to ≤58 mmol/mol. Red = 58 to ≤64 mmol/mol. Grey = 64 to ≤75 mmol/mol. Yellow = > 75 mmol/mol

There is a relatively higher cost reduction in the cohort with starting HbA1c levels of > 48 mmol/mol to ≤58 mmol/mol after 25 years (€6.1 billion) compared to the other cohorts with higher starting HbA1c levels but this may be due to a higher number of people in that cohort. The trajectory of cost reduction reduces between 15 and 25 years in all of the cohorts as the effects of the escalations of treatment in earlier years reduces. Figure 1 shows cumulative cost reduction by starting level of HbA1c.

The improvements in outcomes are demonstrated in an estimated reduction of more than 1.2 million complications cumulatively over 25 years in the intervention group. There is an estimated cumulative reduction in complications of 266,000 after 5 years; 675,000 after 10 years; 974,000 after 15 years and 1.2 million after 20 and 25 years.

**Fig. 2:**
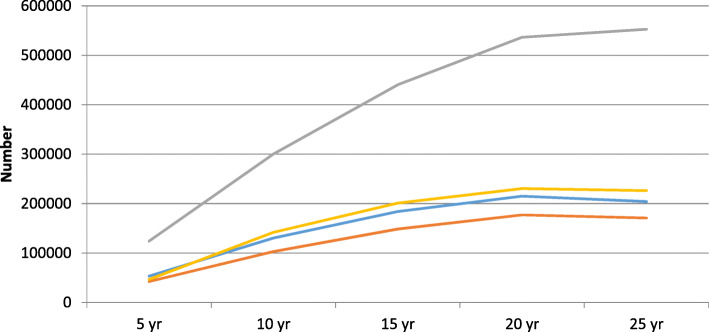
Cumulative reduction of types of complication for the Spanish type 2 diabetes population with starting HbA1c > 48 mmol/mol. Blue = Eyes. Red = Renal. Grey = Feet. Yellow = CVD

More than 550,000 of the avoided complications after 25 years relate to diabetic foot. These include nearly 300,000 avoided ulcers and almost 200,000 cases of neuropathy. Around 200,000 complications relating to eye disease would be avoided after 25 years, including 85,000 cases of diabetic retinopathy. 170,000 renal complications would be avoided over 25 year under the scenarios modelled, including around 13,000 cases of end stage renal disease, which has a considerable cost. Around 226,000 CVD events would be avoided over 25 years, including nearly 60,000 myocardial infarctions. Table 4 and Fig. 2 show the cumulative estimated reduction in complications over 25 years.

**Table 4:** Reduced incidence of complications for the Spanish type 2 diabetes population with starting HbA1c > 48 mmol/mol (per 1000 population)

**> 48 to ≤ 58 mmol/mol**	**5 years**	**10 years**	**15 years**	**20 years**	**25 years**
Eye disease					
BDR	5.5	22.8	33.1	38.8	36.0
PDR	0.3	1.7	3.0	3.9	4.1
ME	5.1	20.7	30.7	36.0	33.5
SVL	0.1	2.4	5.7	8.7	10.6
Cataract	1.8	6.3	8.8	10.2	9.3
*TOTAL*	*12.7*	*54.0*	*81.3*	*97.6*	*93.5*
Renal disease					
MA	8.0	31.0	45.0	52.0	47.3
GRP	1.3	7.7	13.9	19.0	19.2
ESRD	0.2	1.4	2.9	4.5	5.3
*TOTAL*	*9.5*	*40.1*	*61.9*	*75.5*	*71.8*
Diabetic foot					
Ulcer	3.5	23.2	42.0	53.5	52.9
Recurrent ulcer	0.5	9.1	30.7	56.0	75.0
Amputation ulcer	0.2	3.2	8.9	14.5	17.8
Amputation recurrent ulcer	0	0.4	1.6	3.5	5.3
Neuropathy	26.8	85.6	106.9	110.1	94.5
*TOTAL*	*31.0*	*121.6*	*190.2*	*237.5*	*245.4*
Cardiovascular disease					
CHF onset	0.5	5.2	7.7	8.8	8.4
PVD onset	2.4	9.2	13.7	16.5	15.5
Angina	0.5	6.0	9.0	10.1	9.7
Diabetes mortality	0	0.9	3.0	5.7	8.0
Stroke event	−0.1	4.9	6.8	7.2	6.5
Event fatality	1.4	17.4	27.8	32.7	32.9
MI event	0.9	16.7	26.8	31.3	31.3
*TOTAL*	*5.7*	*59.7*	*94.8*	*112.3*	*112.2*
**58 to ≤ 64 mmol/mol**	**5 years**	**10 years**	**15 years**	**20 years**	**25 years**
Eye disease					
BDR	3.4	8.0	10.9	11.4	9.5
PDR	0.3	0.8	1.3	1.5	1.3
ME	3.1	7.7	10.6	11.2	9.3
SVL	0.1	1.1	2.2	3.1	3.4
Cataract	0.9	2.1	2.8	2.9	2.5
*TOTAL*	*7.8*	*19.7*	*27.7*	*30.01*	*26.1*
Renal disease					
MA	4.6	11.2	15.2	15.7	12.6
GRP	0.9	3.1	5.1	6.3	5.5
ESRD	0.1	0.6	1.1	1.5	1.5
*TOTAL*	*5.7*	*14.8*	*21.5*	*23.5*	*19.6*
Diabetic foot					
Ulcer	2.4	8.9	14.5	16.7	15.4
Recurrent ulcer	0.4	4.5	12.0	19.9	24.7
Amputation ulcer	0.2	1.5	3.3	5.0	5.6
Amputation recurrent ulcer	0	0.2	0.7	1.3	1.9
Neuropathy	14.7	29.4	34.7	32.3	26.4
*TOTAL*	*17.7*	*44.5*	*65.02*	*75.2*	*74.0*
Cardiovascular disease					
CHF onset	0.5	1.8	2.4	2.5	2.0
PVD onset	1.3	3.1	4.4	4.5	3.8
Angina	0.5	2.0	2.8	2.8	2.4
Diabetes mortality	0	0.4	1.0	1.7	2.4
Stroke event	0.4	1.7	2.1	2.1	1.7
Event fatality	1.6	6.3	8.7	9.5	8.6
MI event	1.6	6.2	8.4	8.9	8.2
*TOTAL*	*6.0*	*21.6*	*29.9*	*32.1*	*29.2*
**64 to ≤ 75 mmol/mol**	**5 years**	**10 years**	**15 years**	**20 years**	**25 years**
Eye disease					
BDR	6.9	11.5	14.5	16.3	15.4
PDR	0.7	1.3	1.9	2.3	2.3
ME	6.7	11.4	14.6	16.5	15.7
SVL	0.6	1.9	3.4	4.8	5.6
Cataract	1.9	3.2	3.9	4.4	4.1
*TOTAL*	*16.9*	*29.3*	*38.4*	*44.2*	*43.1*
Renal disease					
MA	9.8	16.2	20.4	22.8	21.2
GRP	2.3	4.8	7.3	9.4	9.7
ESRD	0.4	1.1	1.8	2.5	2.9
*TOTAL*	*12.5*	*22.1*	*29.5*	*34.7*	*33.8*
Diabetic foot					
Ulcer	5.9	14.3	20.9	24.8	24.6
Recurrent ulcer	1.6	8.9	20.1	31.3	39.5
Amputation ulcer	0.7	2.7	5.3	7.6	9.1
Amputation recurrent ulcer	0.1	0.5	1.3	2.3	3.4
Neuropathy	31.0	43.2	47.2	47.2	41.1
*TOTAL*	*39.3*	*69.6*	*94.7*	*113.3*	*117.8*
Cardiovascular disease					
CHF onset	1.9	3.0	3.6	3,8	3.6
PVD onset	2.4	4.3	5.7	6.6	6.2
Angina	2.1	3.3	4.0	4.3	4.0
Diabetes mortality	0.2	0.7	1.7	2.7	3.8
Stroke event	1.8	2.7	3.0	3.1	2.9
Event fatality	5.6	9.5	11.6	12.7	12.5
MI event	5.2	8.7	10.4	11.2	10.8
*TOTAL*	*19.1*	*32.2*	*40.0*	*44.4*	*43.8*
**> 75 mmol/mol**	**5 years**	**10 years**	**15 years**	**20 years**	**25 years**
Eye disease					
BDR	6.5	10.7	13.8	15.7	14.6
PDR	0.7	1.3	1.8	2.3	2.3
ME	6.4	10.6	13.8	15.9	14.9
SVL	0.5	1.9	3.4	4.9	5.7
Cataract	1.8	2.9	3.7	4.2	3.9
*TOTAL*	*15.8*	*27.3*	*36.6*	*43.1*	*41.4*
Renal disease					
MA	8.9	14.7	18.8	21.3	19.4
GRP	5.1	10.0	14.9	19.3	22.9
ESRD	0.5	1.2	2.1	3.0	3.4
*TOTAL*	*14.5*	*25.9*	*35.8*	*43.5*	*45.6*
Diabetic foot					
Ulcer	5.6	13.5	20.2	24.5	24.1
Recurrent ulcer	1.4	8.4	19.8	32.0	41.3
Amputation ulcer	0.6	2.6	5.2	7.7	9.4
Amputation recurrent ulcer	0.1	0.4	1.3	2.5	3.7
Neuropathy	28.5	39.7	43.7	43.8	37.0
*TOTAL*	*36.0*	*64.6*	*90.2*	*110.5*	*115.5*
Cardiovascular disease					
CHF onset	1.5	2.4	3.0	3.2	2.9
PVD onset	2.1	3.8	5.3	6.4	5.9
Angina	1.9	3.3	4.0	4.5	4.1
Diabetes mortality	0	0.5	1.6	2.8	4.2
Stroke event	1.4	2.4	2.8	2.9	2.7
Event fatality	4.5	8.1	10.1	11.4	11.1
MI event	4.4	7.7	9.6	10.5	10.0
*TOTAL*	*15.8*	*28.3*	*36.4*	*41.6*	*41.0*

The modelling also shows that over a 25-year period, people receiving the intervention would have a longer time alive and free of complications.

## Discussion

This study shows that people with type 2 diabetes in Spain with raised HbA1c levels could gain significant health and economic benefits through improved glycaemic control. By providing people with treatment at a lower recommended HbA1c, on a population basis, significant numbers of diabetes-related microvascular complications can be avoided and related costs can be reduced. Modelling estimates show that in the population of people with type 2 diabetes, more than 1.1 million complications could be prevented over a 25-year period and more than €14.7 billion could be saved as a result.

Nearly half of the cost reductions observed in this study are attributable to a reduction in complications of the foot (553,000) in the intervention cohort. There were also significant reductions in eye disease (204,000), renal complications (171,000), and cardiovascular disease (226,000) in the cohort with better glycaemic control.

Similar work carried out by Baxter et al., in the UK setting estimated a lower cumulative 25-year cost saving for type 2 diabetes of around €5.1 billion, based on people receiving treatment to HbA1c targets recommended by NICE. In the UK, as in Spain, nearly all of the savings related to reductions in microvascular complications, particularly those for diabetic foot which accounted for around 57% of the estimated cumulative savings.

The potential cost reductions can be seen as the amount of money which could be spent by the Spanish NHS to achieve the overall improvement in glycaemic control, on a population basis, that could result in the scale of avoided complications demonstrated in this study. This does not specify the types of intervention programme that should be adopted, and the way in which the money is spent and the interventions which could be employed to improve glycaemic control will be at the discretion of local health care commissioners.

The types of intervention applicable for individual people will depend on a number of factors and some interventions may have minimal cost. The Spanish primary care RedGDPS algorithm for blood glucose lowering therapy in adults with type 2 diabetes recommends the use of metformin as an initial intervention followed by dual therapy (metformin plus either dipeptidyl peptidase-4 inhibitor, glucagon-like peptide-1, sodium–glucose cotransporter 2 inhibitors or sulfonylurea) and then triple therapy (metformin plus combinations of other therapies), before progression to insulin-based treatment [11]. There will be many variations in the therapies used and associated costs based on the individual requirements of people with type 2 diabetes. The key point is that treatment escalation needs to happen more quickly at the point at which individual people reach the HbA1c threshold of 53 mmol/mol.

The study does though set out the amount of money that could be used in order to achieve improved levels of glycaemic control. It also shows, importantly, that benefits in terms of numbers of complications avoided, and costs saved as a result of reduced numbers of events, are accrued as soon as five years after better control has been achieved. Although there is a slower trajectory for benefits in earlier years, there is much more rapid benefit gain between years 5 and 15.

It is important to note the limitations of this study:

- The study does not include the costs associated with implementing strategies to improve glycaemic control on a population or individual level. There are a broad number of potential interventions that could be used but these will vary dependent on population characteristics and local treatment protocols and infrastructure. Local providers are best placed to determine the optimal use of different interventions for people with type 2 diabetes.
- The sample population used in the economic model are drawn from one area of Spain (Catalonia) but can be considered to be typical primary care patients.
- The study does not take into account any increase in the population with type 2 diabetes. Diabetes prevalence is likely to increase in future years and so, again, these cost reductions may be a relatively conservative estimate.
- Quality adjusted life years have not been considered as this is a budget impact analysis rather than a cost-effectiveness analysis. For the same reason, the future cost reductions have not been discounted by any factor.
- The study does not consider the improvements in quality of life experienced by the intervention group through the avoidance of complications.
- Although the study is based on a validated diabetes model (CDM) predominantly based on the UKPDS studies, it is recognised that management strategies for diabetes may have changed over time.

## Conclusions

Over a 25 year period, Spain could considerably reduce costs and avoid major complications if, on a population level, more ambitious glycaemic control, according to Spanish or EU guidelines, could be achieved among people with type 2 diabetes by reducing the HbA1c threshold for treatment intensification. Although there is a slower trajectory for benefits in earlier years, there is much more rapid benefit gain between years 5 and 15.

## Data Availability

The datasets generated and analysed during the current study are not publicly available due to the fact that they were analysed using the IQVIA Core Diabetes Model but are available from the corresponding author on reasonable request. The database used was http://www.core-diabetes.com/login.asp and this is a closed database which YHEC received permission to access and use for a temporary time period.

## Abbreviations

BDR: Background diabetic retinopathy
CDM: Core Diabetes Model
CHF: Chronic heart failure
GRP: Gross Renal Proteinuria
mmol/mol: Millimoles per mole
MA: Metabolic acidosis
ME: Macular edema
MI: Myocardial infarction
NHS: National Health Service
NICE: National Institute of Health and Care Excellence
PDR: Proliferative diabetic retinopathy
PVD: Peripheral vascular disease
SVL: Severe vision loss
UKPDS: UK Prospective Diabetes Study
